# Integrating economic and health evidence to inform Covid-19 policy in low- and middle- income countries

**DOI:** 10.12688/wellcomeopenres.16380.1

**Published:** 2020-11-19

**Authors:** Anna Vassall, Sedona Sweeney, Edwine Barasa, Shankar Prinja, Marcus R Keogh-Brown, Henning Tarp Jensen, Richard Smith, Rob Baltussen, Rosalind M Eggo, Mark Jit

**Affiliations:** 1 Centre for Health Economics in London, London School of Hygiene & Tropical Medicine, London, UK; 2 Health Economics Research Unit, KEMRI-Wellcome Trust Research Programme, Kenya and Nuffield Department of Medicine, University of Oxford, Oxford, UK; 3 Department of Community Medicine and School of Public Health, Post Graduate Institute of Medical Education and Research, Chandigarh, India; 4 Department of Food and Resource Economics, Faculty of Science, University of Copenhagen, Copenhagen, Denmark; 5 College of Medicine and Health, University of Exeter, Exeter, UK; 6 Radboud University Medical Centre, Radboud University, Nijmegen, The Netherlands; 7 Centre for the Mathematical Modelling of Infectious Disease, London School of Hygiene & Tropical Medicine, London, UK

**Keywords:** Covid, health economics, cost-effectiveness

## Abstract

Covid-19 requires policy makers to consider evidence on both population health and economic welfare. Over the last two decades, the field of health economics has developed a range of analytical approaches and contributed to the institutionalisation of processes to employ economic evidence in health policy. We present a discussion outlining how these approaches and processes need to be applied more widely to inform Covid-19 policy; highlighting where they may need to be adapted conceptually and methodologically, and providing examples of work to date. We focus on the evidential and policy needs of low- and middle-income countries; where there is an urgent need for evidence to navigate the policy trade-offs between health and economic well-being posed by the Covid-19 pandemic.

## Disclaimer

The views expressed in this article are those of the author(s). Publication in Wellcome Open Research does not imply endorsement by Wellcome.

## Introduction

Covid-19 (C19) requires a ‘whole society’ policy response to protect health, economic and social welfare globally. Policy options are multi-sectoral and include scaling-up C19 health services, physical distancing, strengthened social protection and a wide range of additional sectoral, fiscal and macro-economic interventions ^1^. Given the magnitude and breadth of impact of C19, policy action requires careful consideration of trade-offs between the health, economic and social dimensions of population welfare. Understanding, not just the nature, but also the extent of these trade-offs is critical, particularly for low- and middle-income countries (LMICs), to inform C19 policies that maximise overall population welfare during the C19 crisis.

Health policy inevitably involves trade-offs and priority setting between and within different diseases and populations, e.g. between the young vs the old; the severely sick vs the relatively healthy (or less sick); etc. Health sectors employ a range of processes to inform this priority setting ^2^. However, where formal processes for health sector priority setting exist, in the majority of cases they are designed to inform health policy decisions made at the margin; typically appraising the adoption of specific health interventions or technology (referred to as health technology assessment (HTA)) ^3, 4^. Periodically, wider efforts are made to assess whether the range of services included in UHC (health benefit packages) being delivered across the health sector is optimal ^5– 9^.

The field of health economics has a long history of bringing together epidemiological modelling and economics to quantify the trade-offs made both within the health sector and between health and other dimensions of welfare; and supporting the use of such evidence in policy. Cost-effectiveness analysis typically assesses whether population health is maximised within the existing financial constraints (such as a budget limit on healthcare spending): acknowledging that every investment in an intervention within the health sector will mean that another intervention to improve health will be forgone – the concept of ‘opportunity cost’. In priority setting, cost-effectiveness is also considered with other aspects of welfare, for example ensuring equity of health ^10, 11^ or avoiding catastrophic health expenditures ^12, 13^. Health technologies, services or policy interventions are typically assessed from the perspective of the health sector, although sometimes also consider the lost productive time (productivity losses) of individuals who are sick or die prematurely is considered, known as taking a societal perspective ^14^.

We examine here how health economic evidence and more broadly priority setting processes to support decisions around new health technologies (pharmaceutical interventions) and public health policies need to be adapted for C19, focussing on LMICs. We first explore how current approaches may need to be adapted for C19; then we examine the empirical approaches and analytical requirements of providing integrated evidence of health, economic and social impact to health policy makers. We argue for a rapid adaption and application of evidence informed priority setting to ensure that decisions made around C19 policy remain evidence informed, transparent and accountable to the population they serve.

## Broadening the assessment of health sector policy under C19

C19 is no different from any other health issue, in that much of the C19 response will incur ‘within health sector trade-offs’ and therefore it is critical to assess both the impact of overall population health and equity in C19 policy. However, the scale, speed and scope of a pandemic generate impact of exceptional depth and breadth of impact across health systems and, critically, the wider economy, which mean that standard ‘within health sector’ approaches to priority setting need to be extended to ensure that overall allocations to the health sector are optimal, considering all dimensions of population welfare including health, economic and social welfare ^15^. Challenges in doing this, include:

1. The magnitude of the health impact from C19 means that governments may wish to re-evaluate and rapidly change health sector funding levels, in order to maintain some semblance of ‘health as usual’ i.e. current levels of population. Therefore, the **trade-offs may move beyond opportunity costs within the health sector to opportunity costs for other sectors**.
2. **Health, interventions and economic policy goals interact with one another** and are ‘dynamic’ in the sense that ill health impacts on economic and social welfare. Likewise, improvements in economic and social welfare can substantially impact health. For example, reduction in C19 attributable mortality is in part determined by the balance between policies to increase health system capacity and physical distancing. Likewise, economic welfare is in part determined by the balance between physical distancing and social protection policy.
3. C19, and policies such as physical distancing, are likely to have substantial short- to medium-term macro-economic and poverty impacts, so, in order to ensure economic welfare continues ‘as usual’, governments are likely to simultaneously rapidly **alter their ratio of present and future spending and investment policies**.
4. Much of the cost of C19 interventions may be borne at the household level with implications for both household consumption and savings behaviour, labour supply decisions and consequential broader social and health ramifications of catastrophic losses to household income. Even where public expenditures are transferred to households and provide social protection, they eventually have to be paid for from household income, through taxation. Therefore, C19 policy may require **rapid changes to the balance between public sector and private expenditure across the economy.**
5. Many mitigating behavioural responses to C19 **exhibit important externalities,** which means that the actions of each individual directly impact the welfare of others. Typically, this requires a public policy response as individuals will not act in a way that necessarily benefits society as a whole. For instance, patients with mild C19 may not self-quarantine without incentives to do so.
6. C19 policy is also likely to have **substantial distributional impacts**. For, example, there may be trade-offs between costs and benefits incurred by different socioeconomic groups, genders, ethnicities, urban/rural populations, those with differing health status etc. Therefore, heterogeneity of cost and the distribution of health benefits between sub-populations need to be considered.
7. **Responses to C19 are also ‘dynamic’ over time,** for example the ability to exit physical distancing smoothly may depend on the credibility and understanding of government policy during the periods of stringent physical distancing. Likewise, the ability to stimulate improvements in ‘demand-side’ economic behaviour post-crisis may depend on government credibility in handling the economic consequences during the crisis.

In short, informing policy action for C19 is challenging, and needs to include evidence and the analysis of trade-offs within and between health services, but also inform social and economic policies (including financial protection and different sectoral objectives), across population groups, between the public and private sectors, and over time, conducted in an uncertain and rapidly evolving global context. As such, C19 policy making requires additional processes to conventional HTA, evidence generation and review to understanding wide-ranging trade-offs that will be required across a broad range of sectors.

However, before moving evidence to policy challenges demanded by wider inter-sectoral interaction, we examine the generation evidence on the trade-offs incurred within the health sector: between the allocation of resources specifically to C19 services and new technologies versus those for other health conditions. During a pandemic ‘business as usual’ is, or should be, disrupted, but whether it is the exceptional use of bed capacity, or financing of C19 vaccines and treatments, pandemics can impose a clear ‘health-health’ trade-offs.

## Assessing trade-offs across the health sector

Typically, health trade-offs are empirically assessed by establishing whether the implementation of an intervention results in a net improvement in population health. The extent of the ‘opportunity cost’ of C19 expenditure (and hence the net impact on population health) depends on: the extent to which underutilised health service capacity exists and can be employed; the extent to which other health services can be delayed or cancelled without causing harm; the extent of health impact gained from diverting additional funding to the health sector from elsewhere. To conduct these analyses a comparable measure of population health is required, measuring both the extent of both mortality and morbidity impact combined with populations’ preferences for different health states. There is an extensive literature on different ways to measure population preferences for different health states, with many countries using standardised measures such as quality adjusted life years (QALYs) or disability adjusted life years (DALYs) as composite measures of health impact ^16^. Importantly, these measures account for years of healthy life rather than deaths, weighing each year in full health equally. Recent work estimates QALYs and life expectancy gained from saving lives from C19, with life expectancy gained from preventing a C19 death at around 10 years of life in the UK ^17^.

There are no C19 studies, to date, that use QALY/ DALY estimates to examine the optimal balance of public expenditure between those with C19 and those with other health conditions. There is, however, an emerging debate as to whether this is an appropriate trade-off to measure and consider, even within the health sector. In previous pandemics, it has been argued that a ‘rule of rescue’, the moral action to save lives in immediate danger whatever the consequences for other health spend, should be applied ^18^, even if the impact on population is negative. Population preferences around the rule of rescue within the health sector have been investigated, to assist in spending decisions around new life saving technologies, and may be considered during ‘emergency phases’ of pandemics. For example, NICE in the UK, found that most (of a small council of citizens) prioritise immediate life-saving interventions above routine health interventions. However, this prioritisation was only agreed with strict definition of criteria, and even in these circumstances there should some consideration of the extent of impact on population health overall ^18^.

Empirical evidence on the net population health impact of C19 is emerging, suggesting at least in the short term, C19 is incurring substantial opportunity costs for other health areas in LMICs, with health sectors not being able to sufficient address both underlying financial and non-financial constraints in the time required. A study on immunisation in LMICs found that every excess C19 death during routine vaccination would be traded for over 100 deaths in children if routine vaccination ceased ^19^. Concerns have also been raised around the impact of C19 on TB and HIV ^20, 21^. This adds to the evidence from previous epidemics of adverse impacts on malaria; it was estimated that approximately 10,000 additional malaria deaths may have resulted from the cessation of malaria treatment in Guinea, Liberia, and Sierra Leone during the Ebola epidemic ^22^.

C19 stretches existing health sector capacity globally, but early analysis suggests the extent to which C19 services may stretch LMIC financial and health sector capacity is substantial. A recent study estimates that if unmitigated, the total health sector costs for C19 are extremely high, ranging from 58% to 122% of current annual health spend in five LMIC cities ^23^. Barasa *et al.* assessed the capacity of the Kenyan health system to absorb those requiring critical care due to C19 and found that critical bed requirements of C19 surge varied from 12% to 145% of current capacity across different counties in Kenya ^24^. Moreover, hospitals in LMICs typically run at high occupancy and focus on acute care, so displaced services may have a higher opportunity cost than in high income countries (HICs). Even if large temporary hospitals can be constructed, and supplies accessed, qualified health workers will be a critical resource in many LMICs; estimates range from 13 to 56 times current capacity at an unmitigated C19 peak ^19^. While many LMICs have now mitigated the C19, the challenges in some who have not remain substantial, with growing evidence that other critical services are being displaced.

In summary, while emerging evidence suggests within health trade-offs in LMICs will be substantial, at the moment there is little evidence available to assist policy makers respond in a way that considers overall population impact of their choices. There is an urgent need to employ health economic methods at scale to address this evidence gap; especially as new technologies are employed, and determine the optimal allocation resources during the health sector at different stages of the C19 epidemic ^25^.

## Informing C19 policy choice considering trade-offs between health and wider economy

Most empirical evidence to inform C19 policy makers on the extent of the economic impact of C19 are derived from economic models **.** The ‘health economics’ and ‘macroeconomics’ professions apply different models to assess health and economic burdens. Micro-(health) economic models tend to integrate complex epidemiological models, but estimate societal costs by multiplying the time off work with rates of income loss per day off work measured by wages, or sometimes approximated using GDP per capita per day, known as the ‘human capital approach’. They can also be linked with epidemiological models to estimate numbers of households placed below the poverty line as an immediate consequence of illness and health seeking behaviour ^13^.

**Figure 1.**
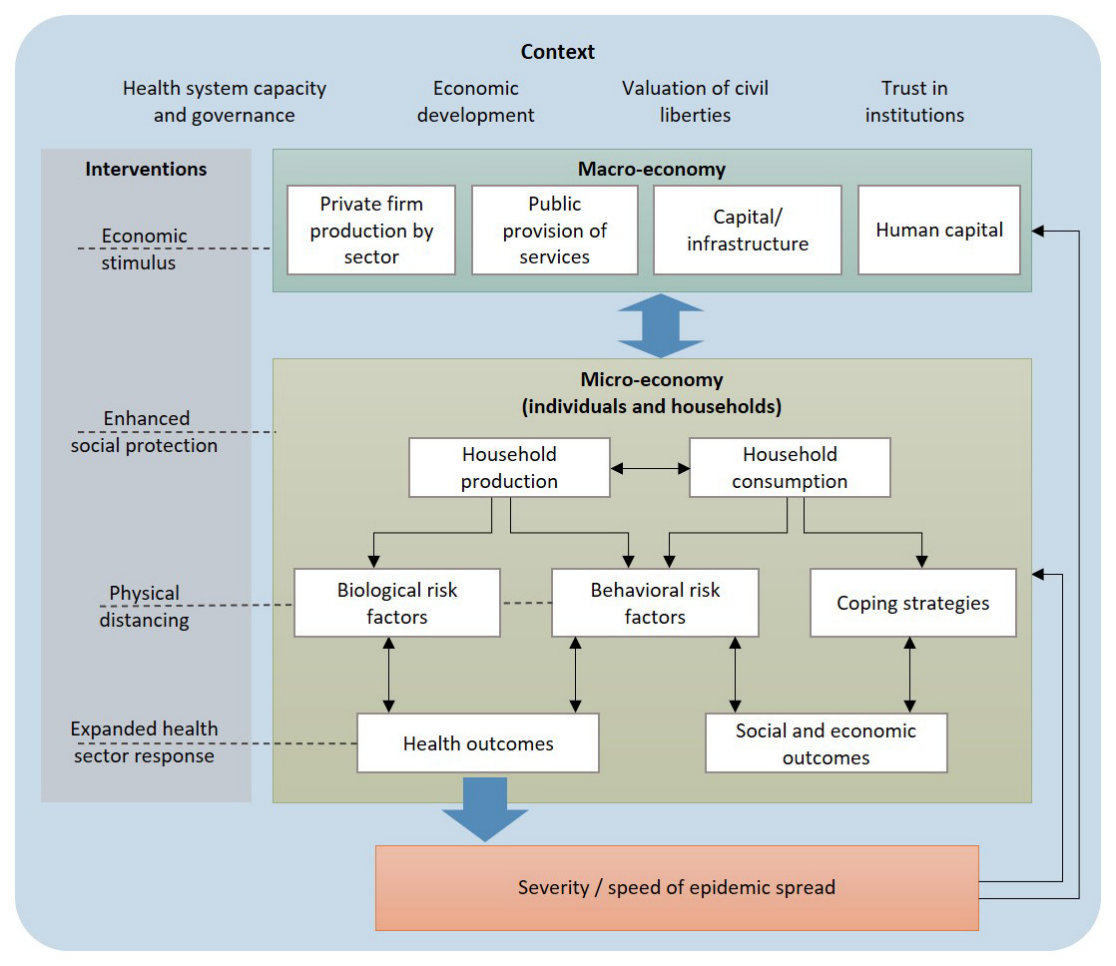
The scope of micro- and macro-economic models of Covid-19.

Conversely, the macro-economic tradition focuses on estimating aggregate economic impact that includes the interactions between individuals and households, employing epidemic estimates produced by external models. Macroeconomic models account for consequential economic behaviour or adjustment, over time, including coping mechanisms such as household decisions to reduce costs and increase savings, workers’ decisions to participate in the workforce, and producers’ and traders’ decisions to maximise profits, to address shocks to the economy (see Figure 1). Importantly, macro-economic models allow for interaction and feedback between different economic sectors, and global macro-economic models are also able to capture the consequences of pandemics on global movement and trade.

There is a long tradition of trying to integrate infectious disease modelling into macro-economic modelling to explore policy trade-offs within one model framework to inform policy makers; and incorporating health to economic to health feedbacks. Work from previous epidemics have included early studies of the macro-economic impact of the HIV pandemic ^26– 30^, which relied on epidemiological projections by World Bank, but no integrated epidemiological model. Recent models employ more elaborate epidemiological models and produce broad ranges of estimates of both health and economic impacts including summary measures in non-monetary units (e.g. symptomatic cases/hospitalizations/deaths averted, work time absenteeism, etc.) and costs (e.g. combined impacts of work absenteeism, health costs, and public mitigation and suppression interventions in local currency units) ^31– 36^. However, there remain challenges in fully integrating the dynamic feedbacks between the progression of infectious diseases and the economy over long time periods. While most macro-economic models of pandemics incorporate the epidemic projections over time, to date only one C19 study captures the feedback between the resulting economic impact to the epidemic trajectory ^37^.

Even where the impacts of different C19 policies can be estimated in an integrated manner, the optimal balance between health and economic policy outcomes is difficult to ascertain as it is inherently value laden. There is some evidence on how populations balance the two outcomes from high income countries (HICs). For example, economists have explored how populations value health and economic welfare using a measure known as the value of statistical life (VSL). VSL estimates the amount individuals would be willing to pay for an improvement in survival, and can be used to convert health impact into monetary value: allowing for an estimate of net welfare ^38^. VSL values are derived from surveys that aim to capture a population’s willingness to pay for a reduction in annual mortality risk, and are reported to range between 20 and 140 times GDP per capita to avoid one death ^39– 41^. Studies in the US applying VSL values to C19 control suggest a net benefit which is higher for social distancing compared to suppression strategies and that social planners should be willing to pay approximately 26% of annual (US) consumption to avoid C19 deaths ^39^. When VSL estimates are extrapolated to LMICs, net benefits that are considerably reduced compared to HICs, although still remain positive ^42^. However, these estimates only point to a general direction as VSL estimates are not generally available for LMIC populations ^42^. Moreover, VSL is controversial, as it is narrowly focussed on mortality and does not consider values around years of life lost and morbidity, and is highly sensitive to individual income. The current evidence base and methods on the valuation of health versus economic welfare in LMICs therefore remains woefully inadequate, and even where models provide joint health and economic outputs, careful decision processes are required to ensure these are weighed correctly (see section on governance below).

### Estimating the impact of C19 without intervention – analytical approaches

**Figure 2.**
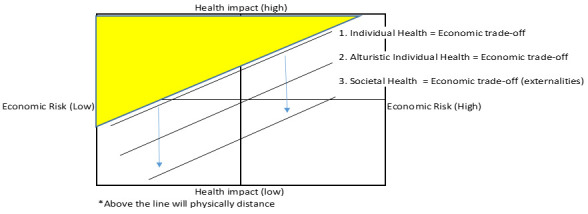
Incremental impact of Covid-19 response on compliance with physical distancing.

The starting point of any empirical analysis of C19 policy on both health and economic welfare is to understand the base case, or the unmitigated C19 impact on the economy or poverty or the ‘cost of illness’. Previous unmitigated pandemics have had substantial economic and poverty impacts. A challenge in estimating the impact of unmitigated pandemics is predicting how individuals judge and act when facing both health and economic risks ^43^. In simple terms, Figure 2 illustrates incremental impact on behaviour of public health policy in the face of C19 risk. Figure 2 divides the population into four groups depending on the level of individual health and economic risk they face. Most of those with a high health risk but little economic risk may voluntarily choose to physical distance (all individuals above line 1). If individuals are altruistic that may increase their propensity to stay in (all individuals above line 2). However, for many either the economic risk will be too high, or the health risk too low, so they may not stay in and spread the disease and harm others (externalities). In this case public health policy may intervene and enforce physical distancing, or alternatively use social protection to reduce individuals’ economic risk, resulting in all individuals above line 3 distancing. The central point being that the economic cost and health impact of the public policy are the costs of moving the line from 2 to 3, and the costs of illness and health impact of C19 are those above line 2.

During the early stages of the HIV pandemic, several macro-economic studies aimed to estimate the costs of illness of HIV in LMICs. Estimates varied by setting, but ranged from approximately 1-5% of GDP ^26– 28^. For example, for Cameroon it was estimated that the loss of skilled workers from HIV would reduce annual economic growth by 1.7% per annum ^44^. Similarly, macroeconomic studies of an unmitigated repeat of the UK flu pandemics from 1957 and 1968 was estimated to be short-lived and, in the worst-case scenario, to constitute a loss of 9.5% of quarterly GDP or a loss of 2.5% of annual GDP in the UK, most of which is due to population coping responses (self-imposed physical distancing) ^32^.

Estimates from the UK suggest that unmitigated C19, with no behaviour change, could result in 490,000 fatalities and impose a direct health-related economic burden of £39.6bn on the UK economy in 2020 (1.7% of GDP), excluding the impact to non-health sectors ^31^. There are no macro-economic studies examining the consequences of an unmitigated epidemic in LMICs. Micro-economic estimates of the cost of illness of C19 suggest that an unmitigated C19 epidemic would incur a substantial cost of illness in the first year ^23^. Households affected by C19 incur costs due to time off work from sickness and incur costs from death. While many C19 deaths occur in older age groups, co-morbidities in working-age (and poor) segments in LMICs may mean that substantial proportions of households may still suffer income losses from an unmitigated C19 epidemic ^23^.

### Assessing the economic impact C19 intervention – analytical approaches

Micro-economic models of C19 ^45^ and initial observational data suggest that the immediate direct impact on poverty of widespread physical distancing or ‘lock down’ will be substantial in LMICs ^46^. Two macro-economic modelling approaches have also been used to date to explore the macro-economic impact of such policies in LMICs. First, aggregate dynamic stochastic general equilibrium (DSGE) models have been used to focus on characterising optimal social planning and explore the balance between economic and health burdens at an aggregate level ^40, 41, 47– 49^. For example, the early use of DSGE models in the US suggested that the scale-up of testing could reduce the economic costs of current lockdown by 2% of GDP ^41^, and that the optimal levels of US teleworking could approach 40% at peak levels ^40^.

Second, multi-sector computable general equilibrium (CGE) models have explored the economic impact of C19 considering interactions between different sectors (e.g. social distancing-related sector-specific business and school closures, and private mitigation behaviours including reduced demand for entertainment, recreational activities, etc.) CGE model applications to infectious diseases are numerous and stretch back to the early stages of the HIV epidemic. The first global CGE applications to respiratory pandemic diseases appeared in the early-2000s, including analyses of SARS ^50^ and pandemic influenza ^51^. Early studies of macro-economic impact in the UK estimated that the period of lockdown would reduce deaths by 95%, but increase the total cost to the UK economy, in 2020, to 29.2% of GDP unless additional mitigating economic policies were put in place to reduce economic co-harms of the suppression strategy ^31^. In comparison, mitigation strategies imposed for 12 weeks would reduce deaths by 29%, but with a total cost of 13.5% of GDP.

Globally a 2.5% drop in GDP is estimated for LMICs, and if C19 continues, in the longer-term LMICs (excl. China) could suffer a 4.8% in GDP ^52^. There is, however, still a dearth of work examining the integrated health and macro-economic impact of different public health policies in LMICs. None of the macro-economic studies for LMICs to date explicitly consider disaggregated impact for different population groups and specifically estimate the numbers of households falling into poverty. Yet, there is substantial evidence from other infectious diseases that suggests this impact may be high. For example, between 27-83% of households affected by tuberculosis in LMICs experience costs that are catastrophic (defined as exceeding 20% of household income) ^53^. Households encountering catastrophic costs due to poor health often respond by adopting coping strategies, which can potentially cause further long term harm, including drawing high-interest loans, selling productive assets (such as livestock), or taking children out of school ^54^.

## The need for context specific evidence from LMICs

Any health economic evidence generated to inform C19 policy needs to reflect the specific structural features of different LMIC economies and health sectors, including the values of specific populations. However, during a pandemic this presents a sizeable challenge given the large numbers of countries involved and limited capacity both to generate and conduct health economic analyses. We therefore identify below the critical and urgent context specific data needs required to inform the applications of modelling efforts outlined above to LMICs. 

### Population characteristics

**Figure 3.**
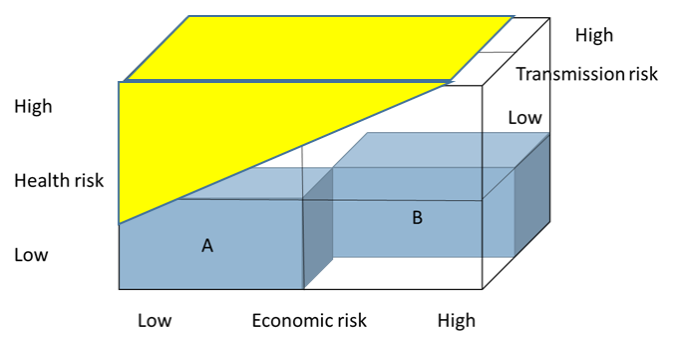
Impact on compliance, including transmission risk.

Fundamentally, any modelling outcomes estimating economic and health trade-offs around the C19 response will be driven by the overlap between populations that are most at risk of infection, at risk of dying, at risk of transmitting C19 or provide most risk to the economy (or vulnerable to income-related shocks). Figure 3 extends Figure 1 to include not just the health risks faced by the individuals, but the risk that they cause harm to others. The yellow shaded area is the population who complies with physical distancing, but do not consider their harm to others. In this example a whole segment of the population who is at high risk of transmission, but has high economic risk and low individual health risk do not comply.

The three dimensions of risk factors are in turn are determined by a combination of biology and social and economic behaviour, that may be correlated (e.g. socioeconomically disadvantaged groups may be more likely to be in poor health and therefore susceptible to severe disease). The challenge for LMIC policy makers, and the scientists/economists who support them, is to better identify these interactions, and develop an understanding that these relationships are dynamic. In other infectious diseases, the relationship between economic risk and these two dimensions of health risk, and the mixing between these populations has been pivotal in how pandemics progress over time, with diseases becoming endemic in populations with high economic risk ^55^.

Estimates of C19 transmission are best made estimated dynamically and predictions of the epidemic curve made by country. C10 transmission models currently rely on population specific demographic data ^56^ and often on non- context specific social contact patterns, using synthetic contact matrices that extrapolate survey data from other settings based on household and societal characteristics ^57^. To estimate and understand both health and economic impact, setting specific data on how social contacts vary by population groups and socio-economic status is urgently required. For example, the probability of being infected by a particular person is likely to be higher for those in close and regular contact (e.g. household contacts) or those in overcrowded working and living conditions; but we know little about how those in these conditions interact with different population groups. 

### Household capacity and resilience

There is also an urgent need to understand the many context specific barriers that prevent individuals, households and firms from complying with C19 policies optimally and may make trade-offs between health and economic welfare more severe, particularly for poor populations. Epidemiological models can also be used to explore and identify between economic risk and disease, but still require primary data to identify explicit links between the specific constraint and disease related behaviour and progression ^58^. Stigma and mental health issues may constrain the ability of individuals to protect themselves. The extent of trust in institutions and social values may influence the extent of adherence to physical distancing. Even when willingness is there, there may be demand side constraints related to economic status, including the affordability and allocation of goods that protect against C19 infection to those most in need, such as basic commodities for hand washing and facemasks. Housing conditions may also be a critical constraint; for example, in many urban informal settlements in LMICs, houses are crowded and one-roomed and have shared and/or outdoor toilets and water sources, making stringent physical distancing impossible.

There are also a wide range of factors that influence the resilience (and reduce the economic risk) of employed households to mitigate economic impacts, including access to loans at affordable interest rates, employment conditions, and the ability to sell assets ^59^. However, for households relying on daily subsistence income, or on foreign remittances, the poverty impact of being unable to work may be severe. While many HIC countries have strong social protection mechanisms in place, many LMICs have weaker mechanisms for providing emergency transfers to populations ^60^. There is a substantial body of economic evidence on the extent to which cash transfers and social protection in LMICs both impact health outcomes and reduce economic risk, which can support governments to design appropriate mechanisms ^58, 61– 65^, and methods such as benefit incidence than can capture the way in which costs and benefits of different public policies impact different population groups ^66, 67^.

### Health sector burden and capacity

Case fatality risks for C19 are also dependent on local health services and the existing disease burden/co-morbidities, which vary substantially by setting and may also be correlated with economic status ^68, 69^. Yes, there is a dearth of information on how different socio-economic groups are accessing both C19 and non-C19 services. In some settings the limiting health system constraint may, however, not be bed capacity, but staff, oxygen, medicines, or protective equipment ^24^. Other elements of capacity such as the strength of the surveillance and testing systems may allow governments to exit physical distancing at an earlier point in the epidemic and reduce economic losses. However, depending on both physical and financial access these may also be less accessible to poorer populations. Previous studies on TB provide an example of how to characterise these constraints ^25, 70^ and explore their impact on infectious disease trajectories and the eventual cost-effectiveness of different interventions ^71^.

## Improving the governance for assessing C19 policies

LMIC governments will need to continuously refine their policies to align with how their populations value health, economic welfare and different dimensions of health. C19 has brought difficult trade-offs, commonly faced by the health sector, to the forefront of public scrutiny. There are several standard frameworks that are typically used to ensure that health policy processes operate in an evidence-based, accountable, and fair manner considering both economic and epidemiological evidence. The analytical frameworks for designing such processes have been developed in the context of HTA in HICs, but also applied in LMICs such as Thailand and Indonesia ^72^.

Table 1 illustrates the types of health and economic trade-offs facing LMICs when making policy choices on social distancing, school closure and expanding health sector capacity to address C19. In their choices, policy makers would ideally maximize public goals such as health, economic welfare and social welfare - yet, in reality, choices impact differently on these goals. Formalised structured decision-making tools such as multi criteria decision analysis (MCDA) can support policy makers in making their choices, and Table 1 provides the starting point of this approach based on the public goals as mentioned in this paper. A complete MCDA would provide a comprehensive overview of the performance of policy choices on all public goals (possibly involving scoring of the performance and weighing of public goals), as input for the policy making process. Yet, given the urgency of C19 response, policy makers may not be able to conduct a complete MCDA and will make the trade-offs in a more deliberative manner ^73^.

**Table 1.** Framework for policy trade-offs in the Covid-19 response (with examples).

Policy choices/ public goals	Health		Economic welfare	Social welfare
	**Impact on Covid-** **19 related health**	**Impact on other** **diseases**		
Physical distancing ^#^	Reduced infections in overall population	Increased mental health problems because of isolation; improved health through improved air pollution	Reduced household income and consumption through reduced tourism, export, foreign direct investment and inflationary pressure	Compromised civil liberties; unrest; food insecurity
School closure	Reduced infections in children; reduced infections in overall population	Increased mental health problems because of isolation	Reduced income if parents have to take time off work without compensation; loss of income for education sector workers if they are not compensated; increased demand for substitutes like online education	Increased exposure of children to violence and exploitation; poor nutrition if children rely on meals provided at schools; stress for teachers for creating and maintaining online learning; challenges measuring and validating learning.
Expanded health sector response	Reduced mortality and morbidity	Treatment delays	Increased health insurance premiums	Displacement of other public expenditure, such as on culture.

# Various physical distancing policies are possible, depending on duration and restrictions. Such policies have different impacts on public goals and could be listed as different policy choices here.

At the core of deliberative approaches is the recognition that policy makers are accountable to the populations they serve and thus need to ensure a legitimate decision-making process. Legitimacy here refers to the reasonableness, or fairness, of policy choices as perceived by stakeholders, which is an important prerequisite for broad societal support for these policies ^73, 74^. For example, the decision whether or not to enforce social distancing should include a consideration of the (potentially competing) interests of people and sectors with varying levels of health and economic vulnerability. Stakeholders are likely to have a wide range of social values and interests that result in different perceptions of what makes particular C19 policy choices valuable, for example limiting the spread of the epidemic, reducing impact on business, and limiting social expenditure. In such processes, stakeholders may reasonably disagree on what values can be used to guide decisions, often explicitly identifying a diverse range of criteria by which to assess policy ^9^.

The combination of the complex mesh of trade-offs, described in previous sections, and the wide range of social values, discussed above, indicates that there is a need for careful deliberative processes in which all stakeholders can meaningfully participate and their values be considered, informed as much as possible by evidence. Such processes should be transparent in the sense that there is clarity between which stakeholders and values are involved, what the available evidence is (and its quality), and how decisions are being taken. The decisions themselves should be made available to the public, including the evidence presented and its argumentation, to ensure public engagement, debate and support for the resulting C19 response. However, given the urgency of the decision-making, such broad consultative processes may not always be feasible and may depend on the stage of the epidemic. At the minimum, policy makers should include key stakeholders representing the health, economic and social dimensions of welfare in its advisory committee to adequately consider all related trade-offs, alongside scientific and economic evidence.

## Conclusion

LMIC policy makers face major challenges in defining their optimal policy response to C19. We call for increased in investment in health economics evidence and evidence informed deliberative policy decisions that consider both health and economic impact. The need is acute in LMICs given the dearth of information and lack of access to both data and joint epidemiological and economic decision support models. There is a risk that countries are forced to rely on qualitative debate, or simple analytical approaches to make decisions, often with severe consequences.

We have highlighted the large body of previous work that can form the basis of that evidence generation to support C19 policy in LMICs, which demonstrates emerging collaboration between economists and epidemiologists, both within the scientific community and the policy arena. Critical priorities include: creating greater capacity (specifically in LMICs) to conduct combined economic and epidemiological modelling and support government decisions; parameterising models with enhanced mapping of social contact data that includes economic status; tracking of the opportunity costs incurred in the health sector, including an improved understanding of the effectiveness of lower cost health sector intervention; economic evaluation to estimate the value of new C19 technologies that fully considers future risk; and communications to support decision makers and the general public understand the uncertainty and evidence quality of current models. However, ultimately, while scientists and academics can generate evidence, and enquire and explore the values of populations, trade-offs between health, populations and the economy require transparent and consultative processes if population welfare is to be protected during the C19 crisis.

## Data availability

No data are associated with this article.
