# Supplementary material for: Integrating economic and health evidence to inform Covid-19 policy in low- and middle- income countries
Source: Wellcome Open Res. 2022 Jul 25;5:272. Originally published 2020 Nov 19. [Version 2] doi: 10.12688/wellcomeopenres.16380.2 (PMC9433912; doi:10.12688/wellcomeopenres.16380.2)
Supplement: Peer review history

# Peer review: Reviewer response for version 2

**DOI:** 10.21956/wellcomeopenres.19889.r51682

**Published:** 2022-08-31

**Authors:** Kalipso Chalkidou

**Affiliations:** 1 Global Health and Development Team, Imperial College London, London, UK; 2 Global Health Policy, Center for Global Development, London, UK

The authors have addressed my comments.

Does the article adequately reference differing views and opinions?

Partly

Are all factual statements correct, and are statements and arguments made adequately supported by citations?

Partly

Is the Open Letter written in accessible language?

Yes

Where applicable, are recommendations and next steps explained clearly for others to follow?

Yes

Is the rationale for the Open Letter provided in sufficient detail?

Yes

Reviewer Expertise:

NA

I confirm that I have read this submission and believe that I have an appropriate level of expertise to confirm that it is of an acceptable scientific standard.

# Peer review: Reviewer response for version 2

**DOI:** 10.21956/wellcomeopenres.19889.r51683

**Published:** 2022-08-09

**Authors:** Beverley Essue

**Affiliations:** 1 Institute of Health Policy, Management & Evaluation, Dalla Lana School of Public Health, University of Toronto, Toronto, ON, Canada

I’m happy with the revisions made and believe this paper is stronger and an important contribution.

Does the article adequately reference differing views and opinions?

Yes

Are all factual statements correct, and are statements and arguments made adequately supported by citations?

Partly

Is the Open Letter written in accessible language?

Yes

Where applicable, are recommendations and next steps explained clearly for others to follow?

Yes

Is the rationale for the Open Letter provided in sufficient detail?

Yes

Reviewer Expertise:

NA

I confirm that I have read this submission and believe that I have an appropriate level of expertise to confirm that it is of an acceptable scientific standard.

# Peer review: Reviewer response for version 1

**DOI:** 10.21956/wellcomeopenres.18017.r43687

**Published:** 2021-06-28

**Authors:** Beverley Essue, Sujata Mishra

**Affiliations:** 1 Institute of Health Policy, Management & Evaluation, Dalla Lana School of Public Health, University of Toronto, Toronto, Canada; 2 Institute of Health Policy, Management & Evaluation, Dalla Lana School of Public Health, University of Toronto, Toronto, ON, Canada

Overall, this is a very pertinent and timely article that discusses the challenges and need to integrate economic evidence with health outcomes and broader outcomes to provide an optimal background for priority setting for Covid-19 (or any pandemic) in a LMIC setting. 

 I would, however, urge that the authors consider pitching this piece as “Integrating economic and health evidence to inform pandemic policy in LMICs” taking Covid-19 as a case study as the lessons are certainly relevant for future pandemic preparedness planning.

 The following comments are suggested to support strengthening the paper:

 Introduction: Paragraph 2

1. Define UHC before using the acronym.

Broadening the assessment of health sector policy under C19: (*overall, this section could benefit from additional references).

1. Acknowledge that both C19 as well its evidence is rapidly evolving and needs urgent attention as the associated mortality and morbidity is relatively high in many LMICs. Thus, the within health-sector trade-offs need to be assessed instantly as compared to any other health issue. A challenge that is unique to the pandemic trajectory.
2. Elaborate on how you think health interventions and economic policy goals interact. In another place, the authors note that they can compete with each other, particularly in LMICs where there are limited resources to be allocated between sectors. Needs citations.
3. With most countries (including LMICs) making progress towards UHCs, how a pandemic like C19 affects government budgets is also a consideration for future planning needs and could be raised here as well.
4. Some countries particularly LMICs with regions with higher population density, it may be impossible to adhere to physical distancing to begin with. Many of the resources including access to water, sanitation and sometimes food etc. are at the community level. How should this context-specific variability be accounted for in the modelling?

Assessing trade-offs across the health sector

 Paragraph 1: Perhaps may be slightly misleading to have this data here. Is there an estimate for LMICs? Health systems in HICs and LMICs are quite different. So, I assume preventing C19 death in LMIC, may have a very different QALY and life expectancy as compared to UK. If the data point remains in the paper, consider adding this explicit caveat.

 Paragraph 2: Is there evidence on how the rule of rescue has been applied during other health emergencies in LMICs? Is it equally applicable in LMICs and HICs? Needs a bit more elaboration.

 Paragraph 4: “While many LMICs have now mitigated the C19, …” I believe the context has changed since this paper was submitted (and this review is overdue!) With multiple waves of C19 and vaccine inequalities, this statement needs revision.

 Informing C19 policy choice considering trade-offs between health and wider economy (*This section would benefit from additional references).

 Paragraph 4: Why does one see this reduction? Is it because LMICs have a lower willingness to pay for each additional life saved?

 Estimating the impact of C19 without intervention – analytical approaches

 Paragraph 1: First, I suggest using perceived health risk instead of a health risk. On most occasions, the true/actual risk (both health and economic) is unknown to the agent/decision maker.

 Second, perhaps this needs more elaboration as to why someone with low economic risk may voluntarily choose to physically distance. I wonder if there may be moral hazard issues particularly if economic cost is lower. For example someone with health insurance, may behave riskier owing to the fact that he/she is protected financially for their risky behaviour.

 Third, authors also need to discuss what happens to people who have higher health risk as well as economic risk (poor people with substantial financial and health burden)? Where do they lie in the figure? Will they also behave riskily? Will a social protection policy help in pushing these people to line 2? 

 Finally, consider adding references to the theory underpinning this figure. Has this figure/idea been used before while modeling cost of pandemic (e.g. HIV)?

 The need for context specific evidence from LMICs

 Population characteristics - Paragraph 1: 

 “Figure 3 extends Figure 1 to include not just the health risks faced by the individuals, but the risk that they cause harm to others.” – I think the Authors mean Figure 2 instead of Figure 1?

 Paragraph 2: I think it is important here to also identify people who have high economic risk and high health risk.

Does the article adequately reference differing views and opinions?

Yes

Are all factual statements correct, and are statements and arguments made adequately supported by citations?

Partly

Is the Open Letter written in accessible language?

Yes

Where applicable, are recommendations and next steps explained clearly for others to follow?

Yes

Is the rationale for the Open Letter provided in sufficient detail?

Yes

Reviewer Expertise:

Health Systems Research, Health economics, Health financing, Equity, Priority setting evaluation.

We confirm that we have read this submission and believe that we have an appropriate level of expertise to confirm that it is of an acceptable scientific standard, however we have significant reservations, as outlined above.

# Response

**Published:** 2022-05-29

**Authors:** Anna Vassall

**Affiliations:** London School of Hygiene & Tropical Medicine (LSHTM) London UK, UK

*We thank both reviewers for their comments, all of which we have addressed and greatly improve the paper.*

 Overall, this is a very pertinent and timely article that discusses the challenges and need to integrate economic evidence with health outcomes and broader outcomes to provide an optimal background for priority setting for Covid-19 (or any pandemic) in a LMIC setting.  I would, however, urge that the authors consider pitching this piece as “Integrating economic and health evidence to inform pandemic policy in LMICs” taking Covid-19 as a case study as the lessons are certainly relevant for future pandemic preparedness planning.

*Thank you for your comments. After a busy period conducting some of the work we originally suggested in this piece at the start of the pandemic, we are now responding to reviewer’s comments. We agree with the above and have made edits that highlight this paper’s broader relevance for future pandemics. While time has moved on, we think it remains useful going forward to map out the concepts and principles around integrating evidence. Without writing a new piece we would like to now ensure this letter is indexed in Medline etc, as part of the body of literature the authors produced during the pandemic. While we add some new references, we have not rewritten this piece as a review of the Covid response – it remains a viewpoint on the factors that should be generally considered in economic evaluation of pandemic responses. During the pandemic we published a living review of the literature integrating health and economics on C19economics.org. We have also recently submitted a systematic review of the models used to integrate health and economic impact used in during the pandemic to another journal, and published an interim review: Saadi N, Chi YL, Ghosh S, Eggo RM, McCarthy CV, Quaife M, Dawa J, Jit M, Vassall A. Models of COVID-19 vaccine prioritisation: a systematic literature search and narrative review. BMC Med. 2021 Dec 1;19(1):318. doi: 10.1186/s12916-021-02190-3.*

**The following comments are suggested to support strengthening the paper:

 Introduction

1. Paragraph 2. Define UHC before using the acronym. *Done*

 

1. Broadening the assessment of health sector policy under C19: (*overall, this section could benefit from additional references). *Done – we have now added further references*

 

1. Acknowledge that both C19 as well its evidence is rapidly evolving and needs urgent attention as the associated mortality and morbidity is relatively high in many LMICs. Thus, the within health-sector trade-offs need to be assessed instantly as compared to any other health issue. A challenge that is unique to the pandemic trajectory. *We agree and have now emphasised the importance of rapid analyses, by adding the following text:*

“Health impact of pandemics such as C19 can rapidly evolve, and therefore **policy responses and the analytics to inform them need to be instantaneous**, despite the complexity involved.”

1. Elaborate on how you think health interventions and economic policy goals interact. In another place, the authors note that they can compete with each other, particularly in LMICs where there are limited resources to be allocated between sectors. Needs citations.

*We agree with this statement and have added the following text:*

**“Health, interventions and economic policy goals interact with one another** and are ‘dynamic’ in the sense that ill health impacts on economic and social welfare. Ill-health can impede the ability to work or exasperate economic inequities. Likewise, improvements in economic and social welfare can substantially impact health and health equity.”

1. C19 affects government budgets is also a consideration for future planning needs and could be raised here as well.

*We agree and have added text:*

 “Therefore, C19 policy may require **rapid changes to the balance between public sector and private expenditure across the economy, having short- and long- term implications for government budgets.**”

1. Some countries particularly LMICs with regions with higher population density, it may be impossible to adhere to physical distancing to begin with. Many of the resources including access to water, sanitation and sometimes food etc. are at the community level. How should this context-specific variability be accounted for in the modelling?

*This is an important point. We now have elaborated with:*

*“*Finally, it is highly likely that both health and economic impacts will heterogeneous across populations, and **responses to pandemics will need to reflect local health, social and economic constraints.”**

 Assessing trade-offs across the health sector

  

1. Paragraph 1: Perhaps may be slightly misleading to have this data here. Is there an estimate for LMICs? Health systems in HICs and LMICs are quite different. So, I assume preventing C19 death in LMIC, may have a very different QALY and life expectancy as compared to UK. If the data point remains in the paper, consider adding this explicit caveat.

*Agreed, we have now changed the reference to one that relates to LMICs, see:*

*“*Estimates of DALYs averted for C19 for LMICs found that disability from C19 would increase DALYs lost by up to a third ^17^^“^.

1. Paragraph 2: Is there evidence on how the rule of rescue has been applied during other health emergencies in LMICs? Is it equally applicable in LMICs and HICs? Needs a bit more elaboration.

*We could not find a discussion of this in specifically related to LMICs. There has been some discussion of prioritising urgent services, those where health would decline substantially if not treated quickly, but we could not find additional material, so did not change the text substantially apart from being clear this work has primarily been considered in respect of HICs.* See Blanchet K, Alwan A, Antoine C, Cros MJ, Feroz F, Amsalu Guracha T, Haaland O, Hailu A, Hangoma P, Jamison D, Memirie ST, Miljeteig I, Jan Naeem A, Nam SL, Norheim OF, Verguet S, Watkins D, Johansson KA. Protecting essential health services in low-income and middle-income countries and humanitarian settings while responding to the COVID-19 pandemic. BMJ Glob Health. 2020 Oct;5(10):e003675. doi: 10.1136/bmjgh-2020-003675. PMID: 33028701; PMCID: PMC7542611.

1. Paragraph 4: “While many LMICs have now mitigated the C19, ...” I believe the context has changed since this paper was submitted (and this review is overdue!) With multiple waves of C19 and vaccine inequalities, this statement needs revision.

*We agree and have deleted this sentence*

 Informing C19 policy choice considering trade-offs between health and wider economy (*This section would benefit from additional references).

1. Paragraph 4: Why does one see this reduction? Is it because LMICs have a lower willingness to pay for each additional life saved?

*Yes, this is partially correct. LMICs have a lower ability to pay (and higher opportunity costs), so may be less willing to pay*

 Estimating the impact of C19 without intervention – analytical approaches

 1. Paragraph 1: First, I suggest using perceived health risk instead of a health risk. On most occasions, the true/actual risk (both health and economic) is unknown to the agent/decision maker.

*We agree and have now edited the text accordingly*

 2. Second, perhaps this needs more elaboration as to why someone with low economic risk may voluntarily choose to physically distance. I wonder if there may be moral hazard issues particularly if economic cost is lower. For example someone with health insurance, may behave riskier owing to the fact that he/she is protected financially for their risky behaviour.

*This is a good point. We are supposing that those with a high health risk and low economic risk (in terms of income effect) may be able to distance, but we do not examine the reasons for that low risk. It could be because they are able to work from home, or for reasons as you say that they are socially protected. We have added a reference (Sweeney et al, that examines this in more detail).*

1. Third, authors also need to discuss what happens to people who have higher health risk as well as economic risk (poor people with substantial financial and health burden)? Where do they lie in the figure? Will they also behave riskily?

*We do not know the answer to this question whether health or economic risk will be the most pivotal. We have edited the text to highlight this specific group, and that this is unclear.*

  

1. Likewise, those facing some of those facing either both high or low risks in both domains may chose not to distance. Will a social protection policy help in pushing these people to line 2? Finally, consider adding references to the theory underpinning this figure. Has this figure/idea been used before while modeling cost of pandemic (e.g. HIV)?

*We have now included text on social protection The figure has not been used in pandemics previously it is our own framing. However, several theoretical economic models have applied the same form, defining a behavioural function that considers both health and economic risk*

 The need for context specific evidence from LMICs

  

1. Population characteristics - Paragraph 1:
  “Figure 3 extends Figure 1 to include not just the health risks faced by the individuals, but the risk that they cause harm to others.” – I think the Authors mean Figure 2 instead of Figure 1? – *Done. Thank you for point this out*

 

1. Paragraph 2: I think it is important here to also identify people who have high economic risk and high health risk. - *Done*

# Peer review: Reviewer response for version 1

**DOI:** 10.21956/wellcomeopenres.18017.r41523

**Published:** 2020-12-11

**Authors:** Kalipso Chalkidou

**Affiliations:** 1 Global Health and Development Team, Imperial College London, London, UK; 2 Global Health Policy, Center for Global Development, London, UK

Timely and well written piece well worth indexing. A few comments on additional refs/points of view (with apologies for self referencing!) below: 

1. In the list (1-7) presented early on a few points on (1) where the *‘health as usual’ i.e. current levels of population" *may need explaining; on (2) where I I wonder if you could make your examples a bit more explicit/obvious? e.g. relationship between poverty and unemployment on one hand and neonatal mortality or preventable deaths? e.g. ^1^; (3-4) Not sure how 3 and 4 are different. 
2. In the assessing trade offs across health sector section, and though you do a lot of this below, I wonder whether you might want to references non COVID health costs of the COVID response. You do cite below work on TB HIV malaria vaccination MCH etc. from LMICs but also work on cancer and other NCDs n HICs - e.g. ^2^ not specific to COVID, ^3^ perhaps also worth citing this and this where these are summed up. Also ^4^ from OECD and this etc.
3. At the end of this section you discuss lack of evidence; and whilst you do introduce the importance of process later on I wonder whether it is the lack of evidence or rather of a process which brings all the evidence together - in a sense if the focus of media/gov is on COVID then the rule of rescue is applied to COVID alone with limited consideration of the invisible costs on other conditions...so we need a process first perhaps? Which may well also boost the evidence base? Again you discuss below but perhaps raise the issue (which is the centre piece of the analysis) early on?
4. Where you discuss micro (health) econ models, is it worth discussing here the trade offs of COVID tech adoption from drugs to tests to the vaccine(s)? E.g. this, ^5^, perhaps also ^6^ and Cam's piece?
5. On VSL - You do discuss the controversy of VSL - in the context of the UK a VSL approach would mean move to a Cancer Drugs Fund for absolutely everything NICE considers. I am not sure how one can put in perspective the magnitude of VSL estimates vs the reality of the NHS (and worse even) a LMIC health budget. Worth doing though.
6. Same section where you discuss health vs economic welfare indeed - I wonder also whether you want to discuss other refs here giving a negative net benefit or offer a more nuanced approach (i.e. suggest cut offs beyond which things cease to be CE etc.) - for HICs and LMICs eg ^7^, this, ^8^^,^^9^ and similar work undertaken in Malawi. I have serious issues with VSL in the UK and LMICs (worse even extrapolating from overestimated UK and US estimates!) to the extent that it is explicitly uninterested in opportunity costs.
7. A lot of the early modelled estimates of HIV impact on economy ended up not materialising through the full cost approach continues to be used as an advocacy tool (albeit not very successfully as far as MOF are concerned) - esp the early modelled impact of loss of HCW from HIV, with the major returns coming in these analyses from longer lives rather than real GDP growth. Worth flagging? This reminded me - not sure if publicly available yet but you know this lit a lot better! ^10^
8. I am not a fan of unmitigated and I wonder whether you might want to discuss "unmitigated" here - I and others discuss this here and I believe it is worth adding a sentence or two on what exactly unmitigated means in the real world and how useful (and indeed how circular) such numbers end up being when used to inform policy OUTSIDE a more holistic in terms of evidence and of values process for which you call.
9. Linked to the above and the 490,000 deaths number, I think the whole issue with the way this outbreak has been dealt with is the over-reliance on single point highly uncertain estimates of impact on health due to COVID as opposed to a balanced approach to costs and benefits of different response options within and beyond health. It is this nuance which is missing when the whole discourse is driven by such estimates. This by Andrew in the FT sets it out nicely.
  So here I would caveat this estimate (which, arguably and based on some of the modellers' own assessments cannot be trusted for anything beyond a two week time horizon ^11^) and also caveat the "prescription" for reducing the R, again modelled out ex ante but when assessed ex post based on (however imperfect) actual observations seem not to have worked that well (lack of evidence is not the same as evidence of things not working but it ought to moderate one's confidence levels!). A few references to consider and consider citing to this effect: ^12^^,^^13^, this, this and even this next one where the authors seem to be arguing against their data - Fig 3 suggests to me one cannot declare NPIs have worked ^14^ and one more ^15^.
10. On use of HTA in LMICs perhaps (and forgive this includes one of mine!) ^16^ or say for India ^17^ and the 2014 HITA resolution by WHA.
11. In Table 1 I would as a minimum add increased mortality and morbidity (to follow on from treatment delays but also delayed presentation etc.) in the column on other health impacts of the policy response. Otherwise the table seems to suggest that the only mortality/morbidity effects come from COVID.
12. On governance worth also citing the WHO WB Gates iDSI CMCC work (which our Thai leads are trying to get published in peer reviewed journal).

Does the article adequately reference differing views and opinions?

Partly

Are all factual statements correct, and are statements and arguments made adequately supported by citations?

Partly

Is the Open Letter written in accessible language?

Yes

Where applicable, are recommendations and next steps explained clearly for others to follow?

Yes

Is the rationale for the Open Letter provided in sufficient detail?

Yes

Reviewer Expertise:

Health policy; health economics.

I confirm that I have read this submission and believe that I have an appropriate level of expertise to confirm that it is of an acceptable scientific standard, however I have significant reservations, as outlined above.

## References

[1] : Aggregate Income Shocks and Infant Mortality in the Developing World. *Review of Economics and Statistics* .2011;93(3) : 10.1162/REST_a_00084 847-856 10.1162/REST_a_00084

[2] : Mortality due to cancer treatment delay: systematic review and meta-analysis. *BMJ* .2020; 10.1136/bmj.m4087 10.1136/bmj.m4087 PMC761002133148535

[3] : The impact of the COVID-19 pandemic on cancer deaths due to delays in diagnosis in England, UK: a national, population-based, modelling study. *The Lancet Oncology* .2020;21(8) : 10.1016/S1470-2045(20)30388-0 1023-1034 10.1016/S1470-2045(20)30388-0 PMC741780832702310

[4] : Health at a Glance: Europe 2020. 10.1787/82129230-en 10.1787/82129230-en

[5] : Economic evaluation of programs against COVID-19: A systematic review. *Int J Surg* .2020;85: 10.1016/j.ijsu.2020.11.015 10-18 10.1016/j.ijsu.2020.11.015 33227532PMC7679235

[6] : Consideration Of Value-Based Pricing For Treatments And Vaccines Is Important, Even In The COVID-19 Pandemic. *Health Aff (Millwood)* .2020; 10.1377/hlthaff.2020.01548 101377hlthaff202001548 10.1377/hlthaff.2020.01548 33211534

[7] : A cost–benefit analysis of the COVID-19 disease. *Oxford Review of Economic Policy* .2020;36(Supplement_1) : 10.1093/oxrep/graa030 S38-S55 10.1093/oxrep/graa030 PMC749978240504226

[8] : LIVING WITH COVID-19: BALANCING COSTS AGAINST BENEFITS IN THE FACE OF THE VIRUS. *National Institute Economic Review* .2020;253: 10.1017/nie.2020.30 R60-R76 10.1017/nie.2020.30

[9] : What cost decisiveness? A cost benefit analysis of the lockdown to contain COVID-19 in India. *medRxiv* .2020; 10.1101/2020.07.07.20148338 10.1101/2020.07.07.20148338

[10] : Trade-offs between Allocation to Health and Other Sectors. *Policy brief 10 of series “Economic Impact of HIV”* .Forthcoming;

[11] : Assessing the performance of real-time epidemic forecasts: A case study of Ebola in the Western Area region of Sierra Leone, 2014-15. *PLoS Comput Biol* .15(2) : 10.1371/journal.pcbi.1006785 e1006785 10.1371/journal.pcbi.1006785 30742608PMC6386417

[12] : Four Stylized Facts about COVID-19.2020; 10.3386/w27719 10.3386/w27719

[13] : A country level analysis measuring the impact of government actions, country preparedness and socioeconomic factors on COVID-19 mortality and related health outcomes. *EClinicalMedicine* .2020;25: 10.1016/j.eclinm.2020.100464 100464 10.1016/j.eclinm.2020.100464 32838237PMC7372278

[14] : The temporal association of introducing and lifting non-pharmaceutical interventions with the time-varying reproduction number (R) of SARS-CoV-2: a modelling study across 131 countries. *The Lancet Infectious Diseases* .2020; 10.1016/S1473-3099(20)30785-4 10.1016/S1473-3099(20)30785-4 PMC758135133729915

[15] : Lockdown measures in response to COVID-19 in nine sub-Saharan African countries. *BMJ Glob Health* .5(10) : 10.1136/bmjgh-2020-003319 10.1136/bmjgh-2020-003319 33028699PMC7542624

[16] : Strengthening health technology assessment systems in the global south: a comparative analysis of the HTA journeys of China, India and South Africa. *Glob Health Action* .2018;11(1) : 10.1080/16549716.2018.1527556 1527556 10.1080/16549716.2018.1527556 30326795PMC6197020

[17] : Institutionalising health technology assessment: establishing the Medical Technology Assessment Board in India. *BMJ Glob Health* .2017;2(2) : 10.1136/bmjgh-2016-000259 e000259 10.1136/bmjgh-2016-000259 29225927PMC5717947

# Response

**Published:** 2022-05-29

**Authors:** Anna Vassall

**Affiliations:** London School of Hygiene & Tropical Medicine (LSHTM) London UK, UK

**REVIEWER 2**

 Timely and well written piece and well worth indexing.

*Thank you for your comments. We have been busy with Covid work, and some time has passed since your review, but hope that the paper remains relevant as part of the broader range of work now conducted during the pandemic. We now have other papers reviewing work done in this area, so did not want to fully update this paper. However, we have made several edits to make this relevant for future pandemics. We are keen that the paper is indexed and have addressed your comments below.*

 A few comments on additional refs/points of view (with apologies for self-referencing!) below:

- In the list (1-7) presented early on a few points on (1) where the *‘health as usual’ i.e. current levels of population" *may need explaining; on (2) where I I wonder if you could make your examples a bit more explicit/obvious? e.g. relationship between poverty and unemployment on one hand and neonatal mortality or preventable deaths? e.g. 1; (3-4) Not sure how 3 and 4 are different.

*Thank you for these references. We have referred now to maternal mortality to point 1 and in point 4, added in employment. It is correct that points 3 and 4 are very related, but we thought good to have one examining the consequences of the division between government and one on household expenditure, and another focusing on the impact on government expenditure over time.  *

- In the assessing trade offs across health sector section, and though you do a lot of this below, I wonder whether you might want to references non COVID health costs of the COVID response. You do cite below work on TB HIV malaria vaccination MCH etc. from LMICs but also work on cancer and other NCDs n HICs - e.g. 2 not specific to COVID, 3 perhaps also worth citing this and this where these are summed up. Also 4 from OECD and this etc.

*Thank you for the references (again!). They are interesting and useful in this response for readers interested in HICs. As this paper focuses on LMICs we only included the ones from LMICs. *

- At the end of this section you discuss lack of evidence; and whilst you do introduce the importance of process later on I wonder whether it is the lack of evidence or rather of a process which brings all the evidence together - in a sense if the focus of media/gov is on COVID then the rule of rescue is applied to COVID alone with limited consideration of the invisible costs on other conditions...so we need a process first perhaps? Which may well also boost the evidence base? Again you discuss below but perhaps raise the issue (which is the centre piece of the analysis) early on?

*We very much agree* *Providing evidence alone, however, is insufficient, and importantly the production of evidence should be matched with ‘evidence based deliberative processes ’that build on current health technology assessment processes but can respond with the rapidity required for pandemics. We have added text to strengthen this point early in the paper.*

- Where you discuss micro (health) econ models, is it worth discussing here the trade offs of COVID tech adoption from drugs to tests to the vaccine(s)? E.g. this, 5, perhaps also 6 and Cam's piece?

*Agree and we have substituted the current reference for reference 6* *to determine the optimal allocation of resources between different diagnostic, treatment, and preventative interventions.*

- On VSL - You do discuss the controversy of VSL - in the context of the UK a VSL approach would mean move to a Cancer Drugs Fund for absolutely everything NICE considers. I am not sure how one can put in perspective the magnitude of VSL estimates vs the reality of the NHS (and worse even) a LMIC health budget. Worth doing though.

*Agree that applying VSL without any empirical estimation from LMICs is limited. We have highlighted this is the text. Unfortunately, we could not think of a quick way of highlighting this concern further, although agree with the general point.*

- Same section where you discuss health vs economic welfare indeed - I wonder also whether you want to discuss other refs here giving a negative net benefit or offer a more nuanced approach (i.e. suggest cut offs beyond which things cease to be CE etc.) - for HICs and LMICs eg 7, this, 8, 9 and similar work undertaken in Malawi. I have serious issues with VSL in the UK and LMICs (worse even extrapolating from overestimated UK and US estimates!) to the extent that it is explicitly uninterested in opportunity costs.

*Agreed.* *We added text at the end of the VSL section and added in reference 9 to this section*

 A lot of the early modelled estimates of HIV impact on economy ended up not materialising through the full cost approach continues to be used as an advocacy tool (albeit not very successfully as far as MOF are concerned) - esp the early modelled impact of loss of HCW from HIV, with the major returns coming in these analyses from longer lives rather than real GDP growth. Worth flagging? This reminded me - not sure if publicly available yet but you know this lit a lot better! 10

*Agree and we have added text. The issue with the initial estimation of HIV impact, is in part related to the issue below, that early estimates (even with HIV) tended to be of unmitigated epidemics with very little knowledge of infectiousness and transmission. It would be wrong to imply that early models got it wrong, they may have got it right given what was known at the time, but it is true to say that some of the forecasts overestimated the impact on African growth that followed (given all the drivers of growth). We could not find the reference below to ascertain which of these reasons (flawed models, uncertainty around disease, uncertainty around the economy, economic policy changes, health policy changes) led to a less catastrophic impact than some originally forecast. *

- I am not a fan of unmitigated and I wonder whether you might want to discuss "unmitigated" here - I and others discuss this here and I believe it is worth adding a sentence or two on what exactly unmitigated means in the real world and how useful (and indeed how circular) such numbers end up being when used to inform policy OUTSIDE a more holistic in terms of evidence and of values process for which you call. Linked to the above and the 490,000 deaths number, I think the whole issue with the way this outbreak has been dealt with is the over-reliance on single point highly uncertain estimates of impact on health due to COVID as opposed to a balanced approach to costs and benefits of different response options within and beyond health. It is this nuance which is missing when the whole discourse is driven by such estimates. This by Andrew in the FT sets it out nicely. So here I would caveat this estimate (which, arguably and based on some of the modellers' own assessments cannot be trusted for anything beyond a two week time horizon 11) and also caveat the "prescription" for reducing the R, again modelled out ex ante but when assessed ex post based on (however imperfect) actual observations seem not to have worked that well (lack of evidence is not the same as evidence of things not working but it ought to moderate one's confidence levels!). A few references to consider and consider citing to this effect: 12, 13, this, this and even this next one where the authors seem to be arguing against their data - Fig 3 suggests to me one cannot declare NPIs have worked14 and one more15.

*This is a contentious issue, that was critical in the early forecasts of Covid impact. While unmitigated estimates are often used in the very initial stages of pandemics to alert policy makers to the extent of any catastrophic impact if nothing is done, they are often made based on highly scarce data, and under considerable uncertainty. Moreover, they cannot be validated as a policy response quickly follows and therefore in reality ‘an unmitigated epidemic cannot be observed’. Moreover, (as your references suggest), while unmitigated epidemics are argued to provide a useful comparator against which to measure the impact of interventions, even without government intervention they are unlikely to occur, as individuals within populations will act, and therefore unmitigated pandemics remain a conceptual construct, rather than a forecast of any real-world consequence of pandemics.*

*In essence, we agree with your concern that early estimates need to be seen and responded to as highly uncertain. However, it is important to also recognise that even if early on pandemics are highly uncertain, it is a risk that is more important to policy, and faced with this policy may be precautionary. Precautionary policies do not necessarily fail to recognise uncertainty but can instead be seen as a response to it.*

- On use of HTA in LMICs perhaps (and forgive this includes one of mine!) 16 or say for India 17 and the 2014 HITA resolution by WHA.

*Agree and have added reference 16. Thank you for this reference.*

- In Table 1 I would as a minimum add increased mortality and morbidity (to follow on from treatment delays but also delayed presentation etc.) in the column on other health impacts of the policy response. Otherwise the table seems to suggest that the only mortality/morbidity effects come from COVID.

*Done*

  

- On governance worth also citing the WHO WB Gates iDSI CMCC work (which our Thai leads are trying to get published in peer reviewed journal).

*We could not find so were not able to add.*
